# Low frequency of knockdown resistance mutation (L1014F) and the efficacy of PBO synergist in multiple insecticide-resistant populations of Anopheles gambiae in Ikorodu, Lagos State, Nigeria

**DOI:** 10.4314/ahs.v23i1.27

**Published:** 2023-03

**Authors:** Fagbohun Ifeoluwa Kayode, Idowu Emmanuel Taiwo, Adedapo O Adeogun, Olagundoye Olalekan, Ikebaku Precious Chimdalu, Orisaheyi Iyanuoluwa Olayilola, Oyeniyi Tolulope Amos, Chinenye Lynda Nkemeh, Olubunmi Adetoro Otubanjo, Yusuf Oladosu, Olubunmi Adetoro Otubanjo

**Affiliations:** 1 Molecular Vector Research Unit, Nigeria Institute of Medical Research; 2 Department of Zoology, University of Lagos; 3 Institute of Tropical Agriculture and Food Security, Universiti Putra Malaysia (UPM), Serdang 43400, Malaysia

**Keywords:** *Malaria*, *Anopheles gambiae*, insecticide resistance, knockdown resistance, metabolic resistance

## Abstract

**Objective:**

We evaluated the susceptibility status of Anopheles gambiae in two communities of Ikorodu, Lagos, Nigeria to DDT, deltamethrin, lambda cyhalothrin and bendiocarb.

**Methods:**

*Anopheles* immature stages were collected from their habitats in the surveyed community and allowed to emerge before exposure adult females to discriminating doses of WHO insecticides including DDT, deltamethrin, lambda cyhalothrin, bendiocarb and malathion. PBO synergistic bioassay was conducted for insecticides where the mosquito samples showed resistance. PCR assay was used for the detection of kdr mutation in the mosquitoes.

**Results:**

Resistance to DDT (40% and 86%) and lambda cyhalothrin (75% and 84%) in Oke-Ota and Majidun respectively. Suspected resistance to deltamethrin (94.9%) and bendiocarb (93.5%) was recorded in Oke-Ota community and the mosquitoes were susceptible to malathion in both communities. KDR mutation (L1014F) from resistance samples from both locations though with a low frequency that significantly departs from Hardy-Weinberg's probability (P> 0.01). PBO synergized bioassay was able to increase knockdown, percentage mortality and restore full susceptibility to deltamethrin and bendiocarb.

**Conclusion:**

Results from this study indicates that the metabolic resistance mechanism is highly implicated in the resistance to different classes of insecticide in Ikorodu and this should be taken into consideration when implementing vector control activities in this area.

## Introduction

Anopheles gambiae is a competent and important vector of malaria and lymphatic filariasis infections in Nigeria. Malaria remains a major cause of infant mortality in sub-Saharan Africa, accounting 25% of infants' mortality in Nigeria^1^,^2^. Malaria is a major cause of hindrance to economic development in many sub-Saharan countries by causing premature death, reduced productivity and huge medical cost.

Malaria control relies largely on the use of insecticides to suppress the mosquito vector population. Therefore, the WHO has a list of approved insecticides to be used in malaria vector control strategies^3^–^5^. Vector control through the use of insecticides has played a major in the global reduction of malaria incidence within the last fifteen years and has been a vital component of several endemic countries' strategic elimination plans^6^–^8^. However, due to the widespread use of insecticides especially for agricultural purposes, Malaria vectors have developed resistance to some of these insecticides^2^,^8^,^9^. This insecticide resistance is associated mainly with reduced target site sensitivity due to a single point mutation in the sodium channel gene, increased metabolic detoxification of insecticides or a combination of both^10^,^11^.

The rapid emergence and spread in insecticidal resistance among the malaria vector population to WHO-approved insecticides presents a challenge to the control of malaria in sub-Saharan Africa^12^,^13^. In Nigeria, insecticides resistance to different classes of insecticides in malaria vectors has been reported in various parts of the country^14^–^16^, the presence of metabolic and knockdown resistance mechanisms has also been reported^17^–^19^.

The aim of this study was therefore to determine the susceptibility status of Anopheles gambiae to selected WHO insecticides of different classes in Ikorodu Local Government of Lagos State, and also evaluate the efficacy of PBO synergist in restoring susceptibility among the resistant populations, the presence of kdr mutation (L1014F) among the resistant population was also investigated.

## Materials and methods

### Study Area and samples collections

This study was conducted in Majidun and Oke-Ota communities of Ikorodu Local Government Area of Lagos State. Ikorodu is a city in Lagos State Nigeria, located North East of Lagos Lagoon. It shares a boundary with Ogun State. Majidun is a community in Ikorodu which is situated between 3°27′E – 3°28′E longitude and 6°37′E latitude and covers about 1.71km^2^ area on the land. Oke-Ota community is situated at latitude: 6.5680678 and longitude: 3.4913443. These communities have several manmade ponds for fish farming that provide favourable breeding sites for mosquitoes, samples of mosquitoes immature stages for this study were obtained from different breeding habitats including; gutters, ponds, small pools of stagnant water, muddy water, and runoff from houses. Anopheline larvae were carefully collected and separated from the culicine larvae, and allowed to emerge in the insectary under standard conditions (temperature: 27oC÷2oC and relative humidity 80%±10%). Emerged adult mosquitoes were fed with only 10% sugar solution.

### Insecticide susceptibility and PBO synergistic bioassays

Insecticide susceptibility tests were carried out using the WHO standard procedures and test kits for adult mosquitoes^20^. The bioassay was conducted using 2-3 days old, glucose fed female Anopheles mosquitoes from the study areas. The WHO susceptibility test kits (WHO tubes and accessories) and Insecticide impregnated papers Bendiocarb (0.1%), DDT (4%), Deltamethrin (0.05%), Lambdacyhalothrin (0.05%), Malathion (5%) and PBO synergist (4%) were used for the bioassays. Each test consists of about 20 to 25 mosquitoes and was carried out in four replicates using the WHO standard protocol. The dead and survived mosquitoes at the end of bioassays were kept separately in well labelled 1.5 mL Eppendorf tubes containing silica gel, for morphological and molecular analyses.

### Morphological and molecular identification

Morphological keys of Gillies and Coetzee^21^ were used in morphological identifications of adult Anopheles mosquitoes. The Anopheles gambiae mosquito was identified mainly by their wings and legs. The genomic DNA of identified Anopheles gambiae were extracted according to the method previously described by Collins et al^22^. Molecular identification of the members of the Anopheles gambiae complex was done using the appropriate primers as described by Collin et al and Fanello et al 22,23.

### Detection of kdr Mutation in Anopheles gambiae collected in Ikorodu

Amplified fragments were using gel electrophoresis and viewed under UV light.

Kdr assay was carried out on the extracted genomic DNA with a little modification to what was described by (24). Using the following primers; Agd1 (5′-ATAGATTCCCCGACCATG-3′), Agd2(5′AGACAAGGATGATGAACC-3′), Agd3(5′-AATTTGCATTACTTACGACA-3′) and Agd4(5′-CTGTAGTGATAGGAAATTTA-3′). Amplified fragments were using gel electrophoresis and viewed under UV light.

### Data Analysis

The knockdown data were subjected to probit analysis to compute the KDT_50_ and KDT_90_ and their 95% confidence intervals. The 24hrs percentage mortality was computed. The susceptibility of Anopheles mosquitoes to insecticides was assessed using the WHO criteria 20: A mortality in the range 98-100% indicates susceptibility, a mortality of 90-97% is suggestive of the existence of resistance and mortality of less than 90% indicates resistance. Allelic frequency and Hardy-Weinberg's probability were also computed. Microsoft Excel and IBM SPSS were used to carry out all statistical analyses.

## Results

The mosquitoes collected and used for this for this study in the study location were identified as Anopheles gambiae s.s. Resistance to DDT was recorded in the two study areas with percentage mortality of 40% and 37%, resistance and possible resistance to pyrethroid was also recorded in the study areas. In Oke-Ota, suspected resistance was recorded for deltamethrin (94.5%) while resistance was reported for lambdacyhalothrin (10.6%). Resistance to both deltamethrin (79.3%) and lambdacyhalothrin (79%) was recorded in Majidun community. For bendiocarb, suspected resistance was recorded in both Oke-Ota (93.5%) and Majidun (94%) communities. Anopheles gambiae s.s. in both communities were susceptible to malathion (Table 1).

**Table 1:** Insecticide susceptibility/resistance status of *Anopheles gambiae* s.l. from Ikorodu Local Government Area, Lagos State

Location	Insecticides	Number exposed (N)	Mortality (%)	KDT_50_ (min)	KDT_95_ (min)	Resistant Status
Oke-Ota	DDT	90	40			Resistant
	Deltamethrin	84	94.9	43.34(40.05±47.4)	115.54(96.12±148.96)	Possible Resistant
	Lambda cyhalothrin	97	10.6	111.04	257.18	Resistant
	Bendiocarb	93	93.5	34.8(29.68±3.61)	57(48.76±79.96)	Possible Resistant
	Malathion	84	98.8	53.92(39.79±132.2)	152.77(83.12±3095.22)	Resistant
Majidun	DDT	86	37			Resistant
	Deltamethrin	87	79.3	64.9 (57.238±77.435)	210.55 (153.817±341.299)	Resistant
	Lambda cyhalothrin	84	79	454.871 (169.235±12669.430)	25856.599 (2339.009±99413755.30)	Resistant
	Bendiocarb	92	94	30.26 (26.973±33.837)	63.795 (53.789±82.473)	Possible Resistant
	Malathion	99	100	53.92(39.79±132.2)	152.77(83.12±3095.22)	Susceptible

**Figure 1:**
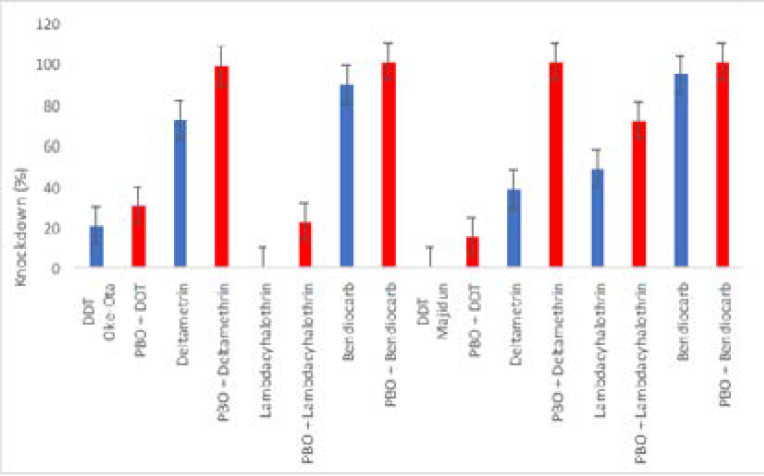
Percentage knockdown at 60 minutes of Anopheles gambiae s.s. from Oke-Ota and Majidun communities of Ikorodu Local Government Area, Lagos State exposed to different insecticides.

**Figure 2:**
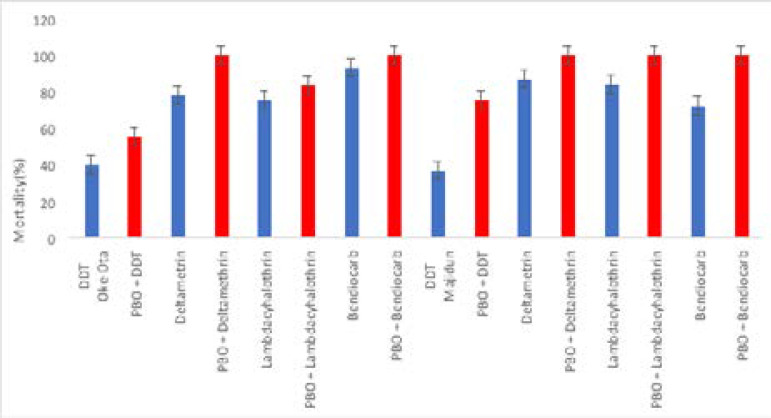
Percentage 24 hours mortality of Anopheles gambiae s.s. from Oke-Ota and Majidun communities of of Ikorodu Local Government Area, Lagos State exposed to different insecticides.

There was an increase in percentage knockdown at different time intervals when resistant s.s. were first exposed to PBO synergist before the insecticide bioassays, over 98% of mosquitoes were knockdown in PBO + deltamethrin and PBO + bendiocarb after 60minutes of exposure in but locations (Figure 1). Result from 24hours post exposure showed that higher percentage mortality was recorded in PBO synergized bioassays compare to the non-synergized ones, full restoration to susceptibility was recorded PBO + lambdacyalothrin from Majidun, PBO + deltamethrin and PBO + bendiocarb from both locations (Figure 2).

KDR mutation (1014F) was detected in a resistant population of Anopheles gambiae in Oke-Ota and Majidun communities in Ikorodu LGA with low allelic frequency that significantly (P< 0.01) departs from the Hardy-Weinberg's value (Table 2).

**Table 2:** Allele frequency of kdr mutation L1014F in Ikorodu Local Government Area, Lagos State

	Insecticide resistance Status	Number examined (N)	Knockdown resistance			Allele frequency	H-W value
			RR	RS	SS		
Oke-ota	Resistant	36	1	6	29	0.11	0.000
	Susceptible	18	1	7	0.06	0.001
Majidun	Resistant	36	2	8	26	0.17	0.000
	Susceptible	18			10		
	

NB: H-W is the probability of the exact test for goodness of fit to Hardy-Weinberg equilibrium; P significant at <0.05. RR: homozygote resistance RS: heterozygote resistance and SS: homozygote susceptible.

## Discussion

Insecticide-based control measures is a major part of malaria vector control therefore, insecticide resistance monitoring should be a vital component of national control efforts. This will ensure that the most effective insecticide is used for control at all times. Insecticide susceptibility/resistance testing helps to determine the efficacy of WHO-approved insecticides in a vector species, in a location at a specific time. In this study, we evaluated the efficacy of different classes of insecticides on An. gambiae s.l. in Ikorodu LGA. Results from this study showed that An. gambiae s.l. from the study locations were resistant to DDT, similar to previous studies in south-western Nigeria and other parts of the country^25^–^29^. Similarly, resistance was also reported in pyrethroid; deltamethrin and lambda cyhalothrin in the study areas. Previous similar studies in Lagos State and other parts of Nigeria have also recorded pyrethroid resistance in various malaria vectors^27^,^28^,^30^,^31^. Cross-resistance to DDT and pyrethroid in malaria vectors has been associated with selection pressure and not well monitor usage of pesticides in agriculture and public health. Pyrethroid remains a major constituent for LLINs and the widely spread resistance to this class insecticide in malaria vectors could be detrimental to the utilizations and efficacy of this tool. Suspected resistance was recorded in An. gambiae to bendiocarb in this study, previous studies have reported similar results in malaria vector in the Southwest and Northern Nigeria^27^,^30^,^32^ though susceptibility was recorded in southeast Nigeria^28^. Carbamates are important in malaria vector control especially for IRS to reduce selection pressure on pyrethroid, the emergence of resistance to this class of insecticide may have a deleterious effect on the control of malaria in the study area.

Recent control efforts have been centred on the development of control strategies that can be effective and efficacious even in the presence of insecticides resistance in malaria vectors. PBO synergist-based insecticides-based intervention measures have been advocated and deployed in different settings where insecticides resistance has been reported^33^–^36^. Results from this study also confirm the importance of PBO synergists in the management of resistant An. gambiae population the Ikorodu LGA. Similarly, previous reports in Nigeria have underscored the importance PBO based insecticide control measures in the areas where insecticides resistance has been recorded in malaria vectors^30^,^37^,^38^.

Despite the level of resistance to DDT and pyrethroid in this study, the level of kdr frequency reported in this study is low, knockdown resistance gene mutation (L1014F) has been associated with DDT and pyrethroid cross-resistance in malaria vectors (24). Conversely, some previous studies in Nigeria and West Africa have reported a high frequency of kdr mutation in highly resistant malaria vectors^37^,^39^–^41^.

## Conclusion

Findings from this study showed that the use of PBO synergists enhances the susceptibility of resistant An. gambiae in the study locations results in this study also indicate that metabolic enzyme activities especially cytochrome P450 mono-oxygenase is involved in the resistance to the pyrethroids and other insecticides tested. Therefore, insecticide-based vector control that incorporates the PBO synergist should be employed when control of An. gambiae s.s is being carried out in these areas.
